# Impact of COVID‐19‐Related Restrictions on Parental Presence and Activities in the Neonatal Intensive Care Unit

**DOI:** 10.1111/ped.70460

**Published:** 2026-07-13

**Authors:** Ryo Itoshima, Arata Oda, Toshimitsu Yanagisawa

**Affiliations:** ^1^ Division of Neonatology Nagano Children's Hospital Azumino Japan; ^2^ Life Science Research Center Nagano Children's Hospital Azumino Japan; ^3^ Department of Clinical Medicine University of Turku Turku Finland

**Keywords:** family‐centered care, pandemic, preterm infants, visitation restriction

## Abstract

**Background:**

This study aimed to evaluate the impact of different restriction measures on parental presence and activities in a neonatal intensive care unit (NICU).

**Methods:**

A prospective cohort study was conducted in a level IV NICU in Japan between February 2021 and October 2023. The study included preterm infants born at < 35 weeks of gestation. Study groups included three different restriction measures: R1, only parents could visit the NICU between 9 a.m. and 3 p.m.; R2, grandparents and parents were allowed; and R3, visiting hours were extended to between 7 a.m. and 9 p.m. Primary outcomes were duration of parental presence (minutes per visit) and visiting frequency (days per week). Linear mixed models were used to adjust for potential confounders and to account for each family.

**Results:**

We analyzed 4589 visits made by 89 families of 110 preterm infants. There were 69, 33, and 24 infants in the R1, R2, and R3 groups, respectively. In a linear mixed model, duration of at least one of the parents' presence in the NICU (mean difference [95% confidence interval]) was significantly longer in the R2 group than that in the R1 group (54.9 [36.9–68.5] min), and also in the R3 group than that in the R2 group (28.4 [9.3–45.8] min). Additionally, the frequency of parental visits to the NICU per week did not change between R1 and R2, but it increased significantly between R2 and R3 (0.5 [0.1–0.9]).

**Conclusions:**

Alleviating restrictive measures in NICUs may increase parental presence and visit frequency.

## 1 Introduction

The presence of parents is essential for preterm infants in neonatal intensive care units (NICUs). Previous studies have shown that the duration or frequency of parental visits to NICUs is associated with better short‐ and long‐term neurodevelopment in preterm infants and better breastmilk feeding rates [1, 2, 3].

The presence of parents in NICUs is also a prerequisite for parents to support their infants and parent‐infant relationship. A meta‐analysis showed that parents' participation in care reduces their stress, lowers the odds of retinopathy of prematurity in their infants, and promotes breastmilk feeding, growth, and neurodevelopment of infants [4]. In 2022, the World Health Organization recommended parental participation in care [5]. Parent‐infant skin‐to‐skin contact is another activity that parents can contribute in NICUs. Meta‐analyses have shown that skin‐to‐skin contact with preterm and/or low‐birth weight infants improve self‐regulation skills later in infancy, shortens length of hospital stay, and reduces mortality [6, 7, 8].

In 2020, the COVID‐19 pandemic occurred, and neonatal intensive care was impacted in many ways. Most NICUs were forced to set up restrictive measures for parental access. Although many NICUs still have some form of restrictive measure in place, few studies have evaluated the impact of such measures on parental presence and participation in care and activities in NICUs. One observational study in Germany showed that eliminating visiting hour restriction due to the COVID‐19 pandemic was not significantly associated with the duration of parental presence in a NICU [9]. The same study also evaluated the association between visiting hour restrictions and the duration of parent‐infant skin‐to‐skin contact; however, other parent activities in NICUs have not been studied. Therefore, this study aimed to evaluate the association between restrictive measures and the duration of parental presence and participation in infant care and activities in a NICU.

## 2 Methods

### 2.1 Design and Participants

This was part of a prospective cohort study comparing three periods with different restriction measures. The study was conducted in a level IV NICU in Japan that functions as a perinatal center of a region. The NICU has 42 neonatal beds, including 24 intensive and 18 step‐down beds, all of which are open‐bay beds. Eligible infants were those born at < 35 weeks of gestation in the study hospital and who required admission into the NICU between February 2021 and October 2023. Infants were excluded from this study if (1) they had any major anomalies on admission, including suspicion of a chromosomal disorder; (2) at least one parent was not Japanese; or (3) their survival was uncertain. Ethical approval was obtained from the Ethics Committee of the Nagano Children's Hospital (S‐02‐68) and written informed consent was obtained from the parents of all participants. The original clinical study was prospectively registered at ClinicalTrials.gov (identifier, NCT04761419).

### 2.2 Restriction Measures

Three restriction measures were implemented during the study period. R1 was implemented from the beginning of the study until July 31, 2022. During R1, both parents visited the NICU between 9 a.m. and 3 p.m. R2 was implemented between August 1, 2022 and May 7, 2023. During the R2 period, up to two parents and grandparents per day were allowed to visit the NICU between 9 a.m. and 3 p.m. R3 was implemented between May 8, 2023 and the end of the study. During the R3 period, up to two parents and grandparents per day were allowed to visit the NICU between 7 a.m. and 9 p.m. Hospitalized postpartum mothers, who were usually hospitalized for 4–7 days, were allowed to visit the NICU for 24 h and 7 days of the week throughout the study period (Table 1).

**TABLE 1:** Details of restriction measures in each period (R1, R2, and R3).

	Visiting hours	Visitors	Postpartum mothers in hospital
R1 (between February 1, 2021 and July 31, 2022)	9 a.m. to 3 p.m. (6 h)	Only parents	24 h and 7 days a week
R2 (between August 1, 2022 and May 7, 2023)		Up to two parents and grandparents	
R3 (between May 8, 2023 and end of the study)	7 a.m. to 9 p.m. (14 h)		

Grandparents often play an important role when infants are admitted to NICUs. First, in Japan, grandparents often help with childcare. Although more fathers have been taking parental leave in recent years, a 2023 survey showed that 46.2% of fathers took parental leave, with a mean duration of 46.5 days [10]. Additionally, it is not recommended for mothers to drive a car in the first postpartum month. Because the study institution is in a rural area and no public transportation was available, the role of grandparents as car drivers could be more important.

### 2.3 Outcome Measures

Primary outcomes were duration of parental presence in the NICU for each visit day (minutes per visit) and frequency of parental visits (days per week). Secondary outcomes were duration of parent‐infant skin‐to‐skin contact, parental caretaking, and interactive activities for their infants on each visiting day (minutes per visit). All outcome measures were considered from the infant's perspective (e.g., having at least one of the parents beside them) and the parent's perspective (e.g., staying beside one of their infants if the parent had twins or triplets).

Duration of the activities was recorded using a modified Parent‐Infant Closeness Diary (Supplement 1). Original diary included three items for each mother and father: presence, holding, and parent‐infant skin‐to‐skin contact [11]. We changed these items to our own settings: parental presence, parent‐infant skin‐to‐skin contact, parental caretaking, and interactive activities for their infants. Permission was obtained from the diary developers to change the items. The diary had 1 day per page and included all four items for both parents. Both parents drew a line on the blank side for the specific items they performed. The start and end times were placed in 5‐min increments.

The presence of parents was defined as being close to their infants. Parent‐infant skin‐to‐skin contact was defined as holding the infant on the parent's bare chest, with only a diaper and cap, if necessary. Caretaking included parent‐infant skin‐to‐skin contact, breastfeeding, bottle feeding, pumping, tube feeding, changing a diaper, wiping the body, bathing, and other essential care for the infant. Interactive activities for the infant included any form of care, hugging, holding, touching, watching, talking, reading books, playing, making something for the infant, writing a diary, and any other activities for the infant. The frequency of parental visits was calculated based on the difference in the number of days between each visit and previous visits.

### 2.4 Statistics

Data were analyzed at each visit. Distributions of the outcome measures are expressed as mean with standard deviation (SD). The Student's *t*‐test was used to compare outcome measures between R1 and R2, and between R2 and R3. Linear mixed models were used to adjust for gestational age, multiple births (singleton vs. twin/triplet), mode of delivery (vaginal vs. cesarean delivery), other children at home (yes vs. no), fathers' parental leave (yes vs. no), and time required from home to the hospital. Some of these were shown to be associated with parental presence in NICUs [12, 13]. Each family was included in the model as a random factor to consider random effects. Group comparisons were expressed as mean difference (MD) with a 95% confidence interval (CI). Transformation of the outcome measures was not necessary in the models because their residuals could be considered normally distributed. All statistical analyses were performed using R statistic software (version 4.3.3) [14] with Tidyverse (version 2.0.0) [15] and lme4 (version 1.1‐31) [16]. Estimated marginal means in the linear mixed models were calculated using Modelbased (version 0.8.9) [17]. Statistical significance was set at *p* < 0.05.

## 3 Results

**FIGURE 1:**
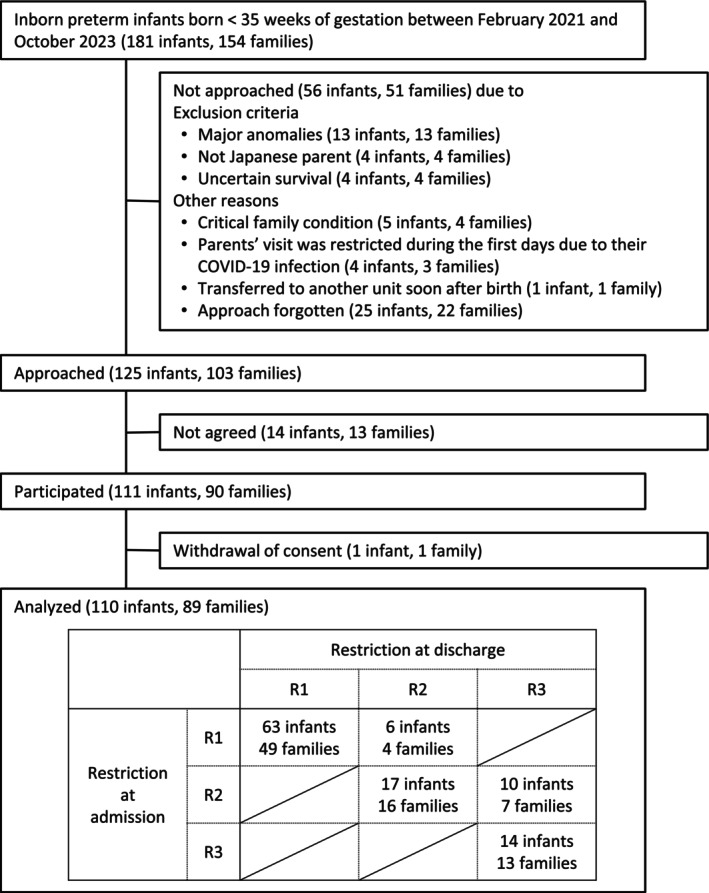
Flow diagram of recruitment and participation and the restriction measures.

A total of 181 preterm infants from 154 families were born at the study institution at < 35 weeks of gestation during the study period. Of 125 infants from 103 families, 111 infants from 90 families agreed to participate. After excluding one infant whose parents withdrew consent, we analyzed 110 infants from 89 families (Figure 1).

Figure 1 shows the number of patients allocated to each restriction group: 69 infants from 53 families in the R1 group, 33 from 27 families in the R2 group, and 24 from 20 families in the R3 group. Some infants and families experienced > 1 restriction during hospitalization. Table 2 summarizes the characteristics of the infants and their parents. These values were comparable between the groups.

**TABLE 2:** Characteristics of the infants and parents in total and in each restriction group.

Infants	Total	R1	R2	R3
	(*n* = 110)	(*n* = 69)	(*n* = 33)	(*n* = 24)
Gestational age, median (IQR), weeks	30.8 (27.3–33.3)	31.0 (27.4–33.1)	30.6 (25.7–33.4)	28.3 (24.7–33.1)
Multiple birth, *n* (%)	39 (35.5)	29 (42.0)	11 (33.3)	2 (33.3)
Cesarean delivery, *n* (%)	76 (72.4)	52 (75.4)	23 (69.7)	12 (63.2)
Apgar score < 7 at 5 min, *n* (%)	30 (28.6)	21 (30.4)	11 (33.3)	7 (36.8)

Parents	Total	R1	R2	R3
	(*n* = 89)	(*n* = 53)	(*n* = 27)	(*n* = 20)
Single parent, *n* (%)	0 (0)	0 (0)	0 (0)	0 (0)
No other children at home, *n* (%)	42 (47.2)	27 (50.9)	12 (44.4)	10 (50.0)
Time required from home to hospital, median (IQR), min	60 (30–70)	60 (30–75)	60 (35–70)	60 (30–60)
Mother's higher education, *n* (%)	31 (35.2)	21 (39.6)	8 (30.8)	7 (35.0)
Father's higher education, *n* (%)	41 (47.1)	21 (39.6)	14 (51.9)	12 (66.7)
Father's parental leave, *n* (%)	26 (29.9)	14 (26.4)	9 (33.3)	4 (22.2)

Abbreviation: IQR, interquartile range.

**FIGURE 2:**
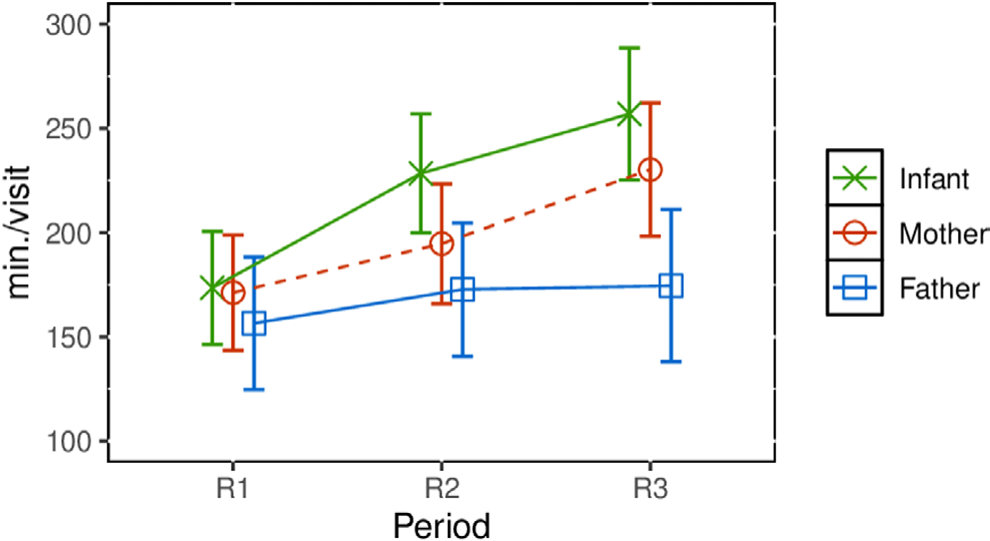
Estimated marginal means (and 95% confidence interval) of parental presence and their changes between R1, R2, and R3 in linear mixed models.

A total of 4589 parental visits were recorded, including 2419, 867, and 1303 in the R1, R2, and R3 groups, respectively. Mean (SD) parental presence was 170.1 (104.8), 181.1 (123.4), and 169.2 (85.7) min per visit in the R1, R2, and R3 groups, respectively, from the infants' perspective. In the linear mixed models, infants had at least one of the parents beside them, which was significantly longer in the R2 group than in the R1 group (MD, 54.9; 95% CI: 36.9–68.5), and in the R3 group than in the R2 group (MD, 28.4; 95% CI: 9.3–45.8). Additionally, the mothers stayed beside their infants significantly longer in the R2 group than in the R1 group (MD, 25.0; 95% CI: 3.1–39.6), and in the R3 group than in the R2 group (MD, 35.0; 95% CI: 14.9–53.7). Duration of fathers' presence did not change significantly (Table 3). Estimated marginal means of parental presence and changes in the linear mixed models are illustrated in Figure 2. Mean (SD) frequency of parental visits was 5.1 (2.2), 5.1 (2.2), and 6.0 (1.8) days per week in the R1, R2, and R3 groups, respectively. In the linear mixed models, infants had at least one of the parents more frequently in the R3 group than in the R2 group (MD, 0.5; 95% CI: 0.1–0.9), but did not change significantly between the R1 and R2 groups. Mothers in the R3 group visited their infants more frequently than those in the R2 group (MD, 0.6; 95% CI: 0.2–1.0), but this did not change significantly between mothers in the R1 and R2 groups. The frequency of paternal visits did not change significantly between the groups (Table 3).

**TABLE 3:** Outcome measures and comparisons between restriction groups.

	Total (*n* = 4589 visits)	R1 (*n* = 2419 visits)	R2 (*n* = 867 visits)	R3 (*n* = 1303 visits)	Estimated marginal mean (95% CI) in LMM, minute			MD (95% CI) in LMM	
					R1	R2	R3	R2 vs. R1	R3 vs. R2
Presence, mean (SD), minute per visit									
Infant	171.9 (103.8)	170.1 (104.8)	181.1 (123.4)^a^	169.2 (85.7)^b^	173.5 (146.4, 200.6)	228.4 (199.9, 256.9)	256.9 (225.2, 288.5)	**54.9 (36.9, 68.5)**	**28.4 (9.3, 45.8)**
Mother	171.6 (103.5)	172.0 (104.0)	166.4 (123.2)	174.3 (87.1)	171.2 (143.5, 198.9)	194.7 (165.9, 223.4)	230.2 (198.3, 262.1)	**23.5 (3.1, 39.6)**	**35.0 (14.9, 53.7)**
Father	155.6 (111.1)	163.5 (123.1)	156.0 (111.5)	140.1 (80.5)^b^	156.5 (124.7, 188.3)	172.7 (140.7, 204.6)	174.5 (138.0, 211.1)	16.1 (−8.6, 37.3)	1.9 (−25.4, 27.9)
Visiting frequency, mean (SD), days per week									
Infant	5.3 (2.1)	5.1 (2.2)	5.1 (2.2)	6.0 (1.8)^b^	5.0 (4.3, 5.7)	4.7 (4.0, 5.4)	5.2 (4.4, 5.9)	−0.3 (−0.6, 0.0)	**0.5 (0.1, 0.9)**
Mother	5.1 (2.2)	4.8 (2.2)	5.1 (2.3)^a^	5.6 (2.0)^b^	4.7 (4.1, 5.3)	4.8 (4.1, 5.4)	5.4 (4.6, 6.1)	0.1 (−0.3, 0.5)	**0.6 (0.2, 1.0)**
Father	3.9 (2.6)	3.7 (2.6)	3.5 (2.4)	4.4 (2.5)^b^	3.7 (2.9, 4.4)	3.9 (3.0, 4.7)	3.6 (2.6, 4.6)	0.2 (−0.4, 0.7)	−0.3 (−0.9, 0.4)
Skin‐to‐skin contact, mean (SD), minute per visit									
Infant	15.5 (36.1)	17.4 (38.6)	12.8 (33.0)	16.3 (36.8)^b^	17.3 (7.9, 26.6)	10.5 (0.9, 20.2)	2.1 (−8.6, 12.7)	**−6.8 (−11.3, −1.7)**	**−8.5 (−14.3, −2.1)**
Mother	14.8 (34.6)	15.7 (35.1)	16.7 (36.0)	12.1 (32.5)^b^	15.4 (9.4, 21.5)	9.5 (2.3, 16.6)	2.6 (−5.5, 10.6)	**−6.0 (−11.0, −0.1)**	**−6.9 (−12.9, −0.3)**
Father	11.2 (31.8)	11.3 (31.6)	13.2 (36.1)	9.8 (28.9)	11.3 (3.7, 18.9)	8.3 (0.0, 16.6)	4.3 (−5.0, 13.7)	−3.0 (−9.2, 3.8)	−4.0 (−11.7, 3.5)
Caretaking, mean (SD), minute per visit									
Infant	66.3 (71.7)	65.8 (79.5)	74.1 (65.6)^a^	62.1 (58.9)^b^	68.2 (45.3, 91.1)	93.7 (70.4, 116.9)	124.8 (99.6, 150.1)	**25.5 (14.6, 34.8)**	**31.1 (18.3, 43.1)**
Mother	70.2 (70.6)	69.0 (74.7)	73.8 (72.2)	70.3 (61.3)	59.6 (38.0, 81.3)	95.6 (71.8, 119.3)	128.3 (102.5, 154.1)	**36.0 (20.5, 47.9)**	**32.7 (18.7, 45.7)**
Father	44.3 (74.8)	49.5 (90.5)	45.2 (58.2)	33.6 (43.5)^b^	48.4 (29.7, 67.2)	52.0 (31.6, 72.5)	47.3 (24.2, 70.4)	3.6 (−10.9, 17.8)	−4.8 (−23.4, 12.1)
Interactive activities, mean (SD), minute per visit									
Infant	144.1 (96.1)	145.5 (101.2)	136.2 (98.4)^a^	146.7 (84.0)^b^	175.2 (147.8, 202.7)	175.2 (147.2, 203.2)	219.5 (188.8, 250.1)	−0.0 (−13.2, 12.3)	**44.3 (27.8, 60.0)**
Mother	154.8 (96.7)	154.2 (98.9)	144.7 (110.3)	162.4 (81.2)^b^	156.1 (125.9, 186.2)	185.1 (154.2, 216.0)	235.8 (202.0, 269.7)	**29.0 (9.2, 44.9)**	**50.8 (31.6, 68.5)**
Father	134.0 (107.3)	145.4 (121.5)	124.4 (97.3)^a^	118.1 (77.7)	142.0 (112.1, 171.9)	148.7 (113.1, 184.4)	155.7 (116.6, 194.7)	6.7 (−18.4, 28.7)	6.9 (−20.3, 33.3)

*Note:* Bold results indicate significant results for the LMM.

Abbreviations: CI, confidence interval; LMM, linear mixed model; MD, mean difference; SD, standard deviation.

^a^ Significant difference between R1 and R2 using the Student's *t*‐test.

^b^ Significant difference between R2 and R3 using the Student's *t*‐test.

The duration and frequency of mothers' presence during the first postpartum week, when mothers in Japan are typically still hospitalized, were also compared. The mean (SD) duration of mothers' presence was 132.6 (77.7), 124.2 (86.3), and 137.4 (75.5) minutes per visit in the R1, R2, and R3 groups, respectively. The mean (SD) frequency of mothers' visits was 6.8 (0.9), 6.8 (0.9), and 6.8 (0.9) days per week, respectively. No significant differences among groups were observed for either outcome in the linear mixed‐model analyses.

The duration of skin‐to‐skin contact received by the infants significantly decreased from R1 to R2 (MD, −6.8, 95% CI: −11.3 to −1.7) and from R2 to R3 (MD, −8.5; 95% CI: −14.3 to −2.1) in the linear mixed model. The duration of skin‐to‐skin contact by mothers also significantly decreased over time but not that by fathers. In contrast, the duration of caretaking significantly increased: from R1 to R2 (MD, 25.5; 95% CI: 14.6–34.8) and from R2 to R3 (MD, 31.1; 95% CI: 18.3–43.1) from the infants' perspective, and from R1 to R2 (MD, 36.0; 95% CI: 20.5–47.9) and from R2 to R3 (MD, 32.7; 95% CI: 18.7–45.7) from the mothers' perspective. The duration of fathers' caretaking did not change significantly. A significant change in the duration of parent‐infant interactive activities was observed from the infants' perspective from R2 to R3 and from the mothers' perspective from R1 to R2 and R2 to R3. The duration of fathers' interactive activities did not change significantly (Table 3).

Intraclass correlation coefficients were also calculated for each model and ranged from 0.10 to 0.29 (see Table S1). For example, in the model comparing the duration of presence measured from the infant's perspective, the variance of the family‐level random intercept was 3250 (SD, 57), whereas the residual variance was 8014 (SD, 90). The corresponding intraclass correlation coefficient was 0.29, indicating that approximately 29% of the total variance was attributable to between‐family differences.

## 4 Discussion

Our study evaluated the impact of changes in NICU restriction measures, including allowing grandparents to visit a NICU and extending their visiting hours. We found that both methods of alleviating NICU restriction measures were associated with mothers' longer presence, shorter duration of mother‐infant skin‐to‐skin contact, and longer caretaking and interactive activities with their infants. The visit frequency of the mothers did not change significantly after allowing grandparents to visit the NICU, but increased after extending the visiting hours. However, fathers' visits and activities did not change after the restrictive measures were alleviated. Mothers' visits and activities may be affected by restrictive measures in a NICU.

Our finding that extending NICU visiting hours was associated with mothers' longer presence contradicts a previous report during the COVID‐19 pandemic [9]. One explanation can be that the duration of parental presence in the baseline cohort is longer in the previous report than that in our study: a mean of 250.8 vs. 172.0 min per visit for mothers. Other statistical issues may have affected the results. Our statistical analyses have better power than those in a previous study because we analyzed the data by visiting day, whereas the previous study analyzed its data by parent. We also found significant improvements in the multivariate analyses that were not included in the previous report. In contrast, our study results were in line with the same previous report showing that extending NICU visiting hours was associated with more frequent visits to NICUs among mothers [9]. Mothers may be more motivated to visit the NICUs if they have sufficient time. Parental longer presence and more frequent visits are not only the basis of their care activities in NICUs, but may also lead to better neurodevelopment of preterm infants [1, 2] and a higher breastmilk feeding rate [3]. Parental presence in NICUs is a fundamental basis of family‐centered care; thus, its principles include 24‐h parental access to NICUs [18]. Our study results support the benefits of extending parental visiting hours and 24‐h parental access to NICUs.

To our knowledge, this was the first study to evaluate how parental presence in NICUs changed after liberalizing the visits of grandparents. Our study showed that the duration of mothers' visits to the NICU increased, but the frequency visits did not. In Japan, allowing grandparents to visit NICUs is thought to be effective in increasing parental presence and visits. Grandparents in Japan are sometimes expected to help parents, especially mothers, by supporting their stay and caretaking in NICUs, as well as giving them rides. Their support is particularly important during the first month postpartum, when mothers are often advised not to drive. This is especially relevant for hospitals such as ours, where access is largely dependent on private cars. The duration of mothers' presence in the NICU increased as expected. The absence of differences during the first postpartum week suggests that later differences in mothers' presence were less likely to be explained by baseline maternal behavioral characteristics. However, our study showed that allowing grandparents to visit NICUs was not sufficient for parents to visit them more frequently. A cohort study in the US showed that grandparents of preterm infants visited the NICUs only 0.4 (SD, 0.7) days per week [3]. In our study, postpartum mothers might have looked for help not from grandparents but from anyone else, such as their husbands/partners or friends. The role of grandparents in NICUs varies, depending on country and culture. Further studies are required to understand whether more appropriate support for parents through grandparents is required.

Fathers' presence and activities in the NICU were not affected by the alleviation of the restriction measures. This result suggests a strong impact of another factor on fathers' presence and activities. The most likely factor may be fathers' parental leave. The proportion of fathers who took their parental leave was 29.9%, and the median [interquartile range] of the duration was 2.0 [1.0–5.5] months in our cohort, which was in line with the national survey [10]. Social initiatives that promote fathers' parental leave are necessary for fathers to visit NICUs more frequently and stay longer. Additionally, the results might have been affected by the difficulty of inviting fathers to the NICU, which is one of the difficult aspects of family‐centered care to implement [19]. Alleviating restrictive measures may not be enough to motivate fathers to visit the NICU.

To our knowledge, this is the first study to show an association between the alleviation of NICU restrictive measures and duration of parental activities in NICUs. We found that allowing grandparents to visit the NICU and extending visiting hours were associated with a longer duration of mothers' caretaking and interactive activities. A longer presence of parents in the NICU may have led to longer care and interactive activities with their infants. In a meta‐analysis, parental participation in NICU care was shown to reduce stress and anxiety among parents, reduce preterm infant length of hospital stay and retinopathy of prematurity, increase their weight, and promote neurodevelopment and breastmilk feeding [4]. Other studies have shown significant associations between mothers' deeper engagement with their infants and better parenting behavior 1 month later [20], and between fathers' deeper engagement with their infants and better psychological well‐being at the age of 16 years [21]. Parental involvement in NICU care was added to the recommendation of the World Health Organization in 2022 [5]. The alleviation of NICU restrictive measures may improve outcomes for infants in NICUs and their parents by promoting parental participation in care and interactive activities.

In contrast, the duration of parent‐infant skin‐to‐skin contact showed an opposite effect in our study. It decreased significantly after liberalizing grandparents' visits and extending visiting hours. We speculate that the implementation of parent‐infant skin‐to‐skin contact may have been strongly affected by the termination of promotional activity approximately a year after the beginning of this study. During the first year of the study period, parent‐infant skin‐to‐skin contact was actively promoted at the NICU of the study institution. A literature review showed the necessity of educational programs to implement and promote parent‐infant skin‐to‐skin contact among preterm infants [22]. Parent‐infant skin‐to‐skin contact may have been more affected by promotional activities than by the alleviation of NICU restrictions. Additionally, changes in restrictive measures may have negatively affected mother‐infant skin‐to‐skin contact. Mothers might have felt reluctant to make skin‐to‐skin contact with their infants if they had other family members with them in the NICU due to embarrassment or time constraints. Mothers might have actively chosen to have their infant skin‐to‐skin contact to make the most of their time with the infant when their meeting time was severely restricted.

During the COVID‐19 pandemic, most NICUs set up restrictive measures for parental access, even though there was little evidence of adverse effects on infants in NICUs and their parents. Our results emphasized the importance of considering both the benefits and disadvantages when establishing restrictive measures in NICUs. Similarly, our study clarified the rationale for parental free access to NICUs as one of the principles of family‐centered care.

We analyzed data from > 4000 visits, and potential confounders were adjusted using linear mixed models. These strengths may have increased the accuracy of the results. The mixed model allowed us to consider the tendencies of each family regarding NICU visits. The moderate‐to‐high intraclass correlation coefficients supported the use of mixed models including family as a random effect. Our study has some limitations. First, it had a non‐randomized design, which may have created differences between cohorts that could not be adjusted for. Second, we did not take into account the impact of the COVID‐19 pandemic itself on the outcome measures. The impact should have been different between the cohorts, as it may have decreased over time after the beginning of the pandemic. Third, the impact of liberalizing the visit of grandparents may differ in big cities, where public transportation is the main means of getting to the hospital.

## 5 Conclusion

Our study showed that allowing grandparents to visit the NICU and extending visiting hours were associated with mothers' longer presence, caretaking, and interactive activities with their preterm infants in the NICU. Mothers' visiting frequency increased only after extending their visiting hours. However, father's presence and activities were not affected by these changes. We need to focus more on the impact and importance of restrictive measures on parents' presence and activities in NICUs.

## Author Contributions

R.I., A.O., and T.Y. contributed to the study concept and design, the acquisition, the interpretation of data, and the manuscript writing. R.I. drafted the study protocol and study registration, carried out the ethical review application, analyses, and the drafting of the initial manuscript. All authors had full access to all the data in the study. A.O. and T.Y. accessed and verified the data. All authors critically revised the manuscript for important intellectual content, approved the final version, and had final responsibility for the decision to submit for publication.

## Funding

This work was supported by the Nagano Children's Hospital (2020‐09) and the Japan Society for the Promotion of Science (JP24K13307).

## Disclosure

The authors have nothing to report.

## Conflicts of Interest

The authors declare no conflicts of interest.

## Supporting information


**Table S1:** Family‐level random‐effect variance, residual variance, and intraclass correlation coefficients for all outcomes in the linear mixed models.

## Data Availability

The data that support the findings of this study are available on request from the corresponding author. The data are not publicly available due to privacy or ethical restrictions.

## References

[1] L. C. Reynolds , M. M. Duncan , G. C. Smith , et al., “Parental Presence and Holding in the Neonatal Intensive Care Unit and Associations With Early Neurobehavior,” Journal of Perinatology 33, no. 8 (2013): 636–641. 23412640 10.1038/jp.2013.4 PMC3700586

[2] R. Latva , L. Lehtonen , R. K. Salmelin , and T. Tamminen , “Visiting Less Than Every Day: A Marker for Later Behavioral Problems in Finnish Preterm Infants,” Archives of Pediatrics & Adolescent Medicine 158, no. 12 (2004): 1153–1157. 15583100 10.1001/archpedi.158.12.1153

[3] L. M. Harris , V. Shabanova , J. L. Martinez‐Brockman , et al., “Parent and Grandparent Neonatal Intensive Care Unit Visitation for Preterm Infants,” Journal of Perinatology 44, no. 3 (2024): 419–427. 37573462 10.1038/s41372-023-01745-x

[4] K. North , R. Whelan , L. V. Folger , et al., “Family Involvement in the Routine Care of Hospitalized Preterm or Low Birth Weight Infants: A Systematic Review and Meta‐Analysis,” Pediatrics 150, no. Suppl 1 (2022): e2022057092O. 10.1542/peds.2022-057092O 35921672

[5] G. L. Darmstadt , N. H. Al Jaifi , S. Arif , and et al , “New World Health Organization Recommendations for Care of Preterm or Low Birth Weight Infants: Health Policy,” EClinicalMedicine 63 (2023): 102155. 37753445 10.1016/j.eclinm.2023.102155 PMC10518507

[6] E. Akbari , N. Binnoon‐Erez , M. Rodrigues , et al., “Kangaroo Mother Care and Infant Biopsychosocial Outcomes in the First Year: A Meta‐Analysis,” Early Human Development 122 (2018): 22–31. 29843051 10.1016/j.earlhumdev.2018.05.004

[7] L. M. Narciso , L. O. Beleza , and A. M. Imoto , “The Effectiveness of Kangaroo Mother Care in Hospitalization Period of Preterm and Low Birth Weight Infants: Systematic Review and Meta‐Analysis,” Jornal de Pediatria 98, no. 2 (2022): 117–125. 34274324 10.1016/j.jped.2021.06.004 PMC9432036

[8] E. O. Boundy , R. Dastjerdi , D. Spiegelman , et al., “Kangaroo Mother Care and Neonatal Outcomes: A Meta‐Analysis,” Pediatrics 137, no. 1 (2016): e20152238. 26702029 10.1542/peds.2015-2238 PMC4702019

[9] R. Schuler , T. Frodermann , M. Waitz , et al., “Effects of Liberalising Visiting Policy and Staff Education on Parental Visiting Duration in the Neonatal Unit,” Acta Paediatrica 113, no. 4 (2024): 684–691. 38226419 10.1111/apa.17106

[10] Ministry of Health Labour and Welfare , National Survey on the Publicly Announced Rate of Male Employees Taking Parental Leave in 2023 (Japanese) (Ministry of Health Labour and Welfare, 2023), https://www.mhlw.go.jp/content/001128241.pdf.

[11] A. Axelin , S. Raiskila , and L. Lehtonen , “The Development of Data Collection Tools to Measure Parent–Infant Closeness and Family‐Centered Care in NICUs,” Worldviews on Evidence‐Based Nursing 17, no. 6 (2020): 448–456. 33210818 10.1111/wvn.12475 PMC7756210

[12] R. Pineda , J. Bender , B. Hall , L. Shabosky , A. Annecca , and J. Smith , “Parent Participation in the Neonatal Intensive Care Unit: Predictors and Relationships to Neurobehavior and Developmental Outcomes,” Early Human Development 117 (2018): 32–38. 29275070 10.1016/j.earlhumdev.2017.12.008 PMC5856604

[13] S. Raiskila , A. Axelin , L. Toome , et al., “Parents' Presence and Parent–Infant Closeness in 11 Neonatal Intensive Care Units in Six European Countries Vary Between and Within the Countries,” Acta Paediatrica 106, no. 6 (2017): 878–888. 28235152 10.1111/apa.13798 PMC5434801

[14] R Core Team , R: A Language and Environment for Statistical Computing (R Core Team, 2025), https://www.R‐project.org/.

[15] H. Wickham , M. Averick , J. Bryan , et al., “Welcome to the Tidyverse,” Journal of Open Source Software 4, no. 43 (2019): 1686.

[16] D. Bates , M. Mächler , B. M. Bolker , and S. C. Walker , “Fitting Linear Mixed‐Effects Models Using lme4,” Journal of Statistical Software 67, no. 1 (2015): 1–48.

[17] D. Makowski , M. S. Ben‐Shachar , I. Patil , and D. Lüdecke , Estimation of Model‐Based Predictions, Contrasts and Means, 2025, https://github.com/easystats/modelbased.

[18] J. M. Roué , P. Kuhn , M. Lopez Maestro , et al., “Eight Principles for Patient‐Centred and Family‐Centred Care for Newborns in the Neonatal Intensive Care Unit,” Archives of Disease in Childhood. Fetal and Neonatal Edition 102, no. 4 (2017): F364–F368. 28420745 10.1136/archdischild-2016-312180

[19] S. Raiskila , L. Lehtonen , B. S. Tandberg , et al., “Parent and Nurse Perceptions on the Quality of Family‐Centred Care in 11 European NICUs,” Australian Critical Care 29, no. 4 (2016): 201–209. 27720034 10.1016/j.aucc.2016.09.003

[20] M. H. Klaus , R. Jerauld , N. C. Kreger , W. McAlpine , M. Steffa , and J. H. Kennell , “Maternal Attachment. Importance of the First Post‐Partum Days,” New England Journal of Medicine 286, no. 9 (1972): 460–463. 5009748 10.1056/NEJM197203022860904

[21] T. Kato , Y. Kachi , M. Ochi , et al., “The Long‐Term Association Between Paternal Involvement in Infant Care and Children's Psychological Well‐Being at Age 16 Years: An Analysis of the Japanese Longitudinal Survey of Newborns in the 21st Century 2001 Cohort,” Journal of Affective Disorders 324 (2023): 114–120. 36566942 10.1016/j.jad.2022.12.075

[22] T. T. Denge , N. E. Bam , W. Lubbe , and A. Rakhudu , “Essential Components of an Educational Program for Implementing Skin‐To‐Skin Contact for Preterm Infants in Intensive Care Units: An Integrative Literature Review,” BMC Pregnancy and Childbirth 24, no. 1 (2024): 281. 38627706 10.1186/s12884-024-06447-6 PMC11022346

