# Supplementary material for: Impact of COVID‐19‐Related Restrictions on Parental Presence and Activities in the Neonatal Intensive Care Unit
Source: Pediatr Int. 2026 Jul 13;68(1):e70460. doi: 10.1111/ped.70460 (PMC13359298; doi:10.1111/ped.70460)
Supplement: Supplementary file 1 — Table S1: Family‐level random‐effect variance, residual variance, and intraclass correlation coefficients for all outcomes in the linear mixed models. [file PED-68-e70460-s001.docx]

**Supplemental Table 1**. Family-level random-effect variance, residual variance, and intraclass correlation coefficients for all outcomes in the linear mixed models.

|  | Random effects | | |
| --- | --- | --- | --- |
|  | Family variance (SD) | Residual variance (SD) | ICC |
| **Presence** | | | |
| Infant | 3250 (57) | 8014 (90) | 0.29 |
| Mother | 1892 (44) | 8751 (94) | 0.18 |
| Father | 1670 (41) | 10273 (101) | 0.14 |
| **Visiting frequency** | | | |
| Infant | 1.22 (1.11) | 3.34 (1.83) | 0.27 |
| Mother | 0.97 (0.99) | 3.62 (1.9) | 0.21 |
| Father | 1.27 (1.13) | 4.67 (2.16) | 0.21 |
| **Skin-to-skin contact** | | | |
| Infant | 188 (14) | 1161 (34) | 0.14 |
| Mother | 128 (11) | 1086 (33) | 0.11 |
| Father | 138 (12) | 895 (30) | 0.13 |
| **Caretaking** | | | |
| Infant | 1404 (37) | 3962 (63) | 0.26 |
| Mother | 1453 (38) | 3737 (61) | 0.28 |
| Father | 521 (23) | 4872 (70) | 0.10 |
| **Interactive activities** | | | |
| Infant | 1968 (44) | 6812 (83) | 0.22 |
| Mother | 2399 (49) | 7148 (85) | 0.25 |
| Father | 1999 (45) | 9118 (95) | 0.18 |

ICC, intraclass correlation coefficient; SD, standard deviation.
